# Saffron Floral By-Products as Novel Sustainable Vegan Ingredients for the Functional and Nutritional Improvement of Traditional Wheat and Spelt Breads

**DOI:** 10.3390/foods12122380

**Published:** 2023-06-15

**Authors:** Débora Cerdá-Bernad, María José Frutos

**Affiliations:** Research Group on Quality and Safety, Agro-Food Technology Department, CIAGRO-UMH, Centro de Investigación e Innovación Agroalimentaria y Agroambiental, Miguel Hernández University, 03312 Orihuela, Spain; dcerda@umh.es

**Keywords:** saffron flowers, polyphenols, antioxidant capacity, bioactive compounds, in vitro digestion, vegan bakery foods, sensory analysis, food waste, upcycling

## Abstract

Saffron (*Crocus sativus* L.) is a traditional Mediterranean plant whose stigmas are used to obtain the most expensive spice in the world. Nevertheless, there is a lack of sustainability in its production, since, to produce 1 kg of saffron, about 350 kg of tepals are discarded. Therefore, this study aimed to develop wheat and spelt breads enriched with saffron floral by-products at a ratio of 0, 2.5, 5, and 10% (*w*/*w*), respectively, and to evaluate their nutritional, physicochemical, functional, and sensory properties, as well as the stability of antioxidant compounds during the in vitro digestion. The results revealed that the addition of saffron floral by-products, especially at 10%, increased the dietary fiber content by 25–30% of traditional wheat and spelt breads; improved their mineral content (270–290 mg/100 g for K, 90–95 mg/100 g for Ca, 40–50 mg/100 g for Mg, and 15–18 mg/100 g for Fe); changed their textural properties; and significantly enhanced the phenolic content and antioxidant ability (at 5 and 10%), which remained stable throughout the in vitro oral and gastrointestinal digestion processes. From a sensory point of view, the addition of saffron flowers modified the organoleptic properties of breads. Thus, these novel vegan enriched breads could exert beneficial effects on human health after their intake, making saffron floral by-products suitable and sustainable ingredients to develop new functional foods such as healthier alternative vegan bakery products.

## 1. Introduction

In recent years, consumers are more aware of the relationship between food and health, considering veganism as a suitable option to change their lifestyle and diet. Therefore, worldwide, vegetarian and vegan diets have become widespread in the population due to greater consumer attention to nutritional, environmental, and ethical aspects [1].

Current trends open up great opportunities for new alternative markets, so food industries are actively seeking the development of novel innovative and sustainable vegan products to meet the needs of consumers. Meat alternatives are dominated by soy derivatives since soy protein is abundant, cheap, and, after hydration, has a meat-like texture, but due to allergies issues, new vegetal sources must be sought [2]. Thus, the recovery and valorization of vegetable by-products from plants and vegetables have become of utmost interest to the food industry. These food by-products are considered high-added value ingredients whose function within the food matrix is to provide health-promoting bioactive compounds. 

**Figure 1:**
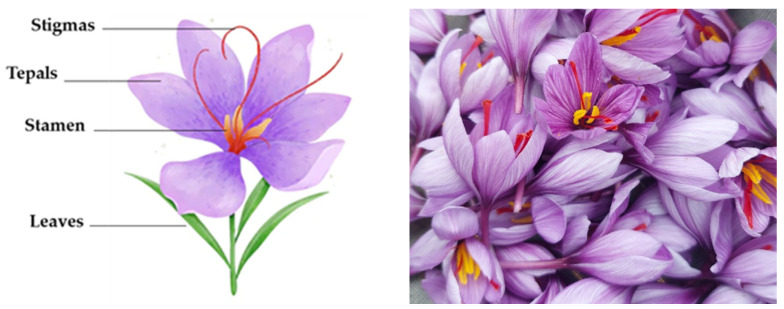
*Crocus sativus* L. flower.

The production of saffron (*Crocus sativus* L.) spice generates large amounts of waste, since only flower stigmas are employed while the rest of the plant is discarded, generating around 350 kg of tepals to obtain 1 kg of the commercial product (Figure 1). However, several studies have reported the potential of these saffron floral by-products at nutritional and biological levels as a source of interesting compounds, such as polyphenols, especially flavonoids (anthocyanins, flavonols), with high antioxidant activity. Their nutritional composition includes carbohydrates (~70%), proteins (~8%), lipids (~4%), fiber (~27%), macro and microminerals, soluble sugars, and vitamin C, among others [3,4,5]. Therefore, the valorization of these saffron floral by-products through their use as novel sustainable vegan ingredients, with high-added value for the production of new functional food products, would lead to the minimization of their environmental impact, in addition to ensuring sustainability and profitability of the saffron industrial sector, developing, at the same time, an alternative vegetal source that would represent a new income for saffron farmers. 

The selection of food matrices as carriers of these plant ingredients is an essential factor to ensure the stability of their functional properties in the final product. Bread, a natural and balanced staple food, which contains a large amount of essential nutrients—carbohydrates, proteins, vitamins A, B1, B2, niacin, folic acid, and minerals—could be considered as a promising carrier matrix of functional substances [6]. Bread has been considered the food par excellence by many civilizations since ancient times, being the result of baking a mixture of flour, water, yeast, and salt. Furthermore, the high consumption and availability of this traditional bakery product, especially wheat and spelt breads, together with its quick and easy production, makes it a suitable matrix for its enrichment with functional ingredients providing bioactive compounds in the diet with positive health effects through the prevention or reduction of the risk of certain diseases [7]. It should be noted that spelt flour was used for its nutritional quality, presenting a high content in protein, and is an excellent source of minerals and vitamin E and B-complex vitamins.

The main aim of this research was to develop different improved traditional formulations of wheat and spelt breads enriched with saffron floral by-products as a sustainable vegan ingredient to produce breads with improved nutritional and functional quality compared to their traditional counterparts. Their physicochemical properties—namely, texture, content of minerals, organic acids, and soluble sugars—and the stability of the antioxidant capacity by ABTS and FRAP assays and total phenolic compounds during the oral and gastrointestinal in vitro digestion, as well as their sensory evaluation, were evaluated. Therefore, this research contributes to the valorization of a currently unexploited biomass through its use as a high-added value ingredient to develop novel functional food products, providing new information regarding the suitability of saffron floral by-products as sustainable alternatives to obtain healthier vegan bakery products.

## 2. Materials and Methods

### 2.1. Raw Materials

Saffron floral by-products, mainly composed of tepals, were obtained from the Castilla-La Mancha region (Spain) after harvesting of saffron flowers and the manual removal of the stigmas during the 2021 harvest season. The fresh flowers were frozen in liquid nitrogen and kept at −80 °C until dehydration in a vacuum oven (VACIOTEM, JP SELECTA^®^, Barcelona, Spain) at 50 ± 3 °C, 28 mbar, for 36 h, and then crushed, sieved (500 μm mesh), and kept at −20 °C until further use. 

For the elaboration of different formulations of bread, two types of flour were used: wheat flour (*Triticum aestivum*; W: 360, P: 100, L: 100, 14.5% of protein) or organic spelt flour (*Triticum spelta*; W:95, P:36, L:105, 13.9% of protein) (El Amasadero, Malaga, Spain). Baker’s active dry yeast (Mauripan, Barcelona, Spain), common commercial salt, and water were also employed. 

### 2.2. Bread Baking Procedure 

The bread formulations were prepared according to a traditional recipe with wheat flour or spelt flour (100%) and adding—based on the weight of flour and following the procedure of Świeca, Gawlik-Dziki, Dziki and Baraniak [8] with slight modifications—water (65–70%), yeast (1%), salt (2%), and dried saffron floral by-products in different concentrations (0%, 2.5%, 5%, and 10%). Eight different formulations were made, four using wheat flour at 0 (WB0%), 2.5 (WB2.5%), 5 (WB5%), and 10% (WB10%) of dried saffron floral by-products; and four formulations using spelt flour at 0 (SB0%), 2.5 (SB2.5%), 5 (SB5%), and 10% (SB10%) of dried saffron floral by-products.

The ingredients were mixed and kneaded for 10 min. A first short fermentation (30 min) was carried out at 30 ± 2 °C and humidity of 40 ± 5%. Loaves were manually round-shaped and left for a second short fermentation (30 min) under the same controlled temperature and humidity conditions. Finally, different bread loaves were baked at 210 °C for 20 min in a domestic oven, the final weight being around 40 g per piece. All formulations were prepared in triplicate. 

Once cooled, a group of breads was stored at −20 °C until further physicochemical analysis, texture, and in vitro digestion tests. Another group was freeze-dried (Christ Alpha 2–4, B. Braun Biotech International, Melsungen, Germany) for 24 h (−25 ± 2 °C, 0.220 mbar). Then, they were crushed, sieved (500 μm mesh), and stored in polyethylene bags at −20 °C until further analysis (minerals, organic acids, and sugar composition).

### 2.3. Physicochemical Characterization

#### 2.3.1. Moisture and Ash

The moisture (%) of fresh samples was determined by keeping bread formulations in an oven at 105 ± 5 °C until they reached constant weight, according to the AOAC [9]. The ash content (%) was carried out by incineration of samples obtained after moisture in a muffle oven (Habersal PR 1300 PAD, Barcelona, Spain) at 550 ± 25 °C, for one hour [9].

#### 2.3.2. pH, Acidity, TSS, aw, Colur

The pH, acidity, TSS, aw, and color were determined according to Cerdá-Bernad, Valero-Cases, Pastor, Frutos and Pérez-Llamas [10], with some modifications. The pH and titratable acidity (expressed as % citric acid) were measured using an automatic titrator (TitroMatic Crison pH-Matic 23, Barcelona, Spain). The determination of total soluble solids (TSS) was carried out with a digital refractometer (Hanna^®^ HI 96801, Bedfordshire, UK) and expressed as °Brix. Fresh samples were previously mixed with distilled water (1:10, *w*/*v*) with an ULTRA-TURRAX^®^ (IKA T18, Werke GmbH & Co, Staufen, Germany) at 5000 rpm for 10 s.

The water activity (aw) of the different fresh samples was determined using a water activity meter (Novasina AW Sprint TH 500, Pfäffikon, Switzerland) at room temperature.

The color was measured with a Minolta CR-300 Chroma Meter (Japan) colorimeter, using the L*, a*, b* scale (CIELAB system). The results were expressed as luminosity L*, a* (greenness/redness), and b* (blueness/yellowness). 

### 2.4. Texture 

Texture profile analysis was performed on bread slices (2.5 cm width) using a TA-XTPlus Texture Analyser (Stable Micro Systems Ltd., Godalming, UK) according to García-Segovia, Igual and Martínez-Monzó [11], a cylindrical aluminum probe (SMS P100, 10 cm in diameter), and a 50 kg load cell. The parameters of the assay were defined as crosshead speed 1.70 mm/s and 40% deformation of the original length. The textural parameters’ hardness (N), cohesiveness, springiness (mm), gumminess (N), and chewiness (N) were determined. 

### 2.5. Minerals

The minerals’ composition was determined according to Serrano-Díaz, Sánchez, Martínez-Tomé, Winterhalter and Alonso [4], with slight modifications. Freeze-dried bread samples weighing 0.5 g were digested with 10 mL of 65% HNO_3_ (*v*/*v*) using a microwave reactor digestor (CEM Mars one, Matthews, NC, USA) for 30 min with a temperature ramp whose final temperature was 200 °C. All samples were filtered (Whatman qualitative filter paper 90 mm), diluted with ultrapure deionized water 1:50 (*v*/*v*), and stored at 4 °C. Total concentrations of macronutrients (Ca, Mg, Na, and K) and micronutrients (Zn, Cu, Mn, and Fe) in the previously mineralized samples were quantified with an Inductively Coupled Plasma Mass Spectrometer (ICPMS-2030, Shimadzu, Kyoto, Japan). Internal standards included calcium (44Ca), magnesium (26Mg), sodium (23Na), potassium (39K), zinc (66Zn), copper (65Cu), manganese (55Mn), and iron (56Fe), and the calibration curves used for quantification showed good linearity (R^2^ ≥ 0.998). The results were expressed as mg/100 g of dw (dry weight).

### 2.6. Organic Acids, Soluble Sugars, and Inulin Content

The extractions were prepared using ultrapure water and a freeze-dried sample/water ratio 1:20 (*w*/*v*). The extracts were shaken for 5 min at 300 rpm on a magnetic stirrer at room temperature (Ovan, mod. MultiMix Heat D-MMH30E, Barcelona, Spain), sonicated for 15 min, and centrifuged at 11,200× *g* for 10 min at 4 °C. The supernatants were filtered (0.45 µm PTFE filter, Millipore, Spain) and stored at −20 °C. 

The identification and quantification of sugars, inulin, and organic acids were carried out by high-performance liquid chromatography using Hewlett-Packard HPLC series 1100 equipment equipped with a Supelcogel C-610H column (30 cm × 7.8 mm) and a Supelcoguard C-610H pre-column (5 cm × 4.6 mm) (Sigma Aldrich, St. Louis, MO, USA). The organic acids were measured at 210 nm in UV-Vis with a diode array detector (DAD G1315A). For sugars, a refractive index detector (G1362A RID) was used. As a mobile phase, 0.1% orthophosphoric acid was used with an injection volume of 20 μL and the flow rate of 0.5 mL/min under isocratic conditions, following the methodology described by Cerdá-Bernad, Valero-Cases, Pastor, Frutos and Pérez-Llamas [10]. The results were expressed as mg/100 g of dw.

### 2.7. In Vitro Digestion

The oral phase of in vitro digestion was performed according to Gawlik-Dziki, Dziki, Baraniak and Lin [12] with slight modifications. Fresh bread samples (2 g) were homogenized in the presence of 30 mL of PBS (phosphate buffered saline) in a stomacher for 30 s to simulate mastication. The solution was previously adjusted to pH 6.75 and alpha-amylase (E.C. 3.2.1.1.) was added to obtain 100 U per mL of enzymatic activity.

Gastrointestinal digestion was carried out following the methodology described by Cerdá-Bernad, Valero-Cases, Pastor, Frutos and Pérez-Llamas [10] for 180 min at 37 °C under continuous stirring. Aliquots were taken after 60 min of gastric digestion (pH = 3), and at 60 min and 120 min of intestinal digestion (pH = 7). All samples were filtered (0.45 μm; Millipore, Spain) and stored at −20 °C until further analysis (antioxidant capacity and total phenolic content).

### 2.8. Antioxidant Properties and Total Phenolic Content (TPC)

The antioxidant capacity determined by using the Ferric Reducing Antioxidant Power (FRAP) method and the 2,2′-Azinobis (3-ethylbenzothiazoline-6-sulfonic acid) (ABTS) radical scavenging method was carried out as described by Cerdá-Bernad, Clemente-Villalba, Valero-Cases, Pastor and Frutos [3]. Briefly, the FRAP reagent was prepared by mixing 300 mM acetate buffer (pH 3.6), 10 mM 2,4,6-tris(2-pyridyl)-s-triazine (TPTZ) solution in 40 mM HCl, and 20 mM FeCl_3_·6H_2_O solution in a volume ratio of 10:1:1, respectively. The absorbance was measured at 593 nm and Trolox (10 mM) was used as the standard solution (0.01–5.00 mM). ABTS radical was prepared mixing ABTS (7 mM) with potassium persulfate (2.45 mM), reacting for 16 h in the dark at room temperature and diluting the solution with ultrapure water until its absorbance was adjusted to 0.70 ± 0.02 at 734 nm. Trolox (10 mM) was used as a reference standard (0.20–3.00 mM). The results were expressed as mmol Trolox/100 g of bread. 

The TPC was determined using the Folin–Ciocalteu methodology as described by Cerdá-Bernad, Clemente-Villalba, Valero-Cases, Pastor and Frutos [3]. Briefly, 100 μL of the different digested samples were mixed with 400 μL of PBS (50 mM) at pH 7.8 and 2.5 mL of Folin–Ciocalteu reagent previously mixed with ultrapure water 1:10 (*v*/*v*). After 2 min, 2 mL of Na_2_CO_3_ (75 g/L) were added and kept at 50 °C for 10 min. The absorbance was measured at 760 nm and the results were expressed as mg of gallic acid equivalents/100 g of bread.

### 2.9. Sensory Analysis 

A quantitative descriptive analysis (QDA) of selected bread formulations was performed by a sensory panel composed of 12 trained judges (five males and seven females) aged between 21 and 60 years, at the Miguel Hernández University (Spain), following the procedure of Sicari, Romeo, Mincione, Santacaterina, Tundis and Loizzo [13] with some modifications. All the panel members had neither allergies nor food intolerances and were regular consumers of bakery products. The samples were labeled with an alphanumeric code and distributed in a random order. A total number of 17 sensory descriptors were considered regarding the appearance, flavor, and texture, using a 10-point scale (Table S1).

### 2.10. Statistical Analysis

Results were expressed as mean ± standard deviation. All experiments were carried out in triplicate. The mean comparisons were undertaken using an analysis of variance (ANOVA) and by the Tukey multiple range test, using the SPSS version 21.0 software package (SPSS Inc., Chicago, IL, USA). Principal Component Analysis (PCA) was conducted using XLSTAT (Microsoft Corp., Washington, DC, USA). The significant differences were established as (*p* ≤ 0.05). 

## 3. Results and Discussion

### 3.1. Characterization of Bread Formulations Enriched with Saffron Floral By-Products: Physicochemical, Nutritional, and Technological Properties

#### 3.1.1. Physicochemical Parameters and Minerals’ Content

Physicochemical and technological properties of the novel bread formulations were evaluated in order to study the influence of adding different amounts of dried saffron floral by-products compared to the control sample. 

Table 1 shows the results of the physicochemical parameters (moisture, ash, pH, acidity, aw, and TSS) of the experimental wheat and spelt bread formulations with saffron floral by-products. Moisture is a relevant factor in the quality of bread since it is related to the shelf life but also influences the texture of the final product. Moisture content regulates the firming rate of bread since its decrease accelerates the process of cross-linking of proteins and starch, which further accelerates the process of firming [14]. 

Regarding the results, in the spelt bread formulations (SB), no statistically significant differences were found between the spelt samples with different concentrations of saffron floral by-products formulations, showing values around 30% of moisture. In the wheat bread samples (WB), moisture values were between 25 and 32%, indicating statistically significant differences between WB2.5% (25.47 ± 2.49%) and WB10% (32.44 ± 2.44%). Moreover, water activity (aw) is also related to the firming process in the bakery products. The addition of different concentrations of dried saffron flowers’ powder did not affect aw compared with the control bread in both wheat and spelt bread formulations since no statistically significant differences were found. These results were similar to those reported in other studies, in which breads enriched with 5 and 10% of insect-based proteins (*Alphitobius diaperinus*) were developed, showing aw values between 0.877 and 0.894 [11]. Therefore, the addition of a new sustainable vegan ingredient to improve the functional properties of spelt and wheat bread did not change the moisture and water activity compared to the control samples. 

As expected, the addition of saffron floral by-products in wheat and spelt bread increased the ash values which are related to the average level of mineral components, since saffron flowers are good sources of minerals [4]. For wheat formulations, the addition of 5% of saffron flower ingredient (3.44 ± 0.10%) significantly increased the ash content with respect to the control sample, WB0% (2.75 ± 0.07%). However, in spelt breads, the ash content (3.37 ± 0.11%) in the formulations with 2.5% of the saffron flower ingredient was significantly higher than the control, SB0% (3.05 ± 0.15%). Furthermore, as shown in Table 2, the macromineral and micromineral concentrations revealed that the enrichment process of wheat and spelt breads with saffron floral by-products had a nutritional positive effect due to an increase in the content of beneficial minerals. 

The most abundant mineral that presented in all wheat and spelt breads was Na due to the salt used in the formulation (600–800 mg/100 g), and no significant differences were shown between bread formulations with different concentrations of saffron floral by-products. Potassium was the macromineral also found in high levels, with increasing concentrations as the amount of saffron floral by-products added was higher, showing that WB10% and SB10% had the highest K content (277 ± 14 and 289 ± 18 mg/100 g, respectively). These values represent 7.91% and 8.26%, approximately, of the Recommended Daily Intake (RDI) of potassium in adults (3500 mg per day) with a consumption of 100 g of WB10% or SB10%, respectively [15]. Moreover, calcium and magnesium were also present in significantly higher amounts than the control bread in those with 5 or 10% of saffron floral by-products added, representing a vegan ingredient that improves the nutritional value of traditional spelt and wheat breads because of its rich mineral composition. The WB10% and SB10% samples showed a concentration of Ca of 91.63 ± 10.02 and 94.56 ± 3.14 mg/100 g, respectively, representing 9.65% and 9.95%, approximately, of the RDI of calcium in adults (950 mg per day) with a consumption of 100 g of wheat or spelt bread with saffron floral by-products ingredient at 10% [15]. Regarding Mg content, WB10% and SB10% had 41.61 ± 1.93 and 48.46 ± 8.03 mg/100 g, respectively, indicating around 13.87% and 16.15% of the RDI of magnesium in adults (300 mg per day), respectively, with a consumption of 100 g wheat or spelt bread with 10% of saffron flowers [15]. These results were in accordance with Fahim, Sadat, Janati and Feizy [16] who reported a high content of K and Ca in saffron petals.

In addition to the macrominerals content, which is relevant for human physiological functions, the microminerals iron, zinc, and manganese were also present in the different bread formulations although in a lower amount compared to Ca, Mg, Na, and K (Table 2). The most abundant micromineral was Fe in all samples, followed by Mn and Zn. The WB10% and SB10% samples showed significantly higher levels of Fe (15.87 ± 1.25 and 17.85 ± 0.40 mg/100 g, respectively) with respect to bread formulations enriched with 2.5 or 5% of saffron floral by-products (WB5%, WB10%, SB5%, SB10%), and with respect to the control samples (WB0%, SB0%). The consumption of 100 g of WB10% and SB10% provides the RDI in adults (11 mg per day) [15]. The amount of Mn in the control samples (1.03 ± 0.19 mg/100 g for WB0% and 0.74 ± 0.09 mg/100 g for SB0%) was statistically significantly lower than WB10% and SB10%, at 1.42 ± 0.21 and 1.37 ± 0.20 mg/100 g, respectively. With respect to Zn content, no statistically significant differences were found in both the wheat and spelt bread formulations, showing concentrations ranging from 0.77 to 1.03 for WB and from 0.71 to 0.96 for SB. 

Minerals are essential micronutrients for human health and wheat flour is an important source of these components, such as Ca (100–200 mg/100 g), Mg (100–200 mg/100 g), Fe (1–5 mg per 100 g), Zn (1–5 mg/100 g), and Cu (0.1–1 mg/100 g) [17]. In addition, saffron floral by-products can provide mineral enrichment through their use in these novel vegan bread formulations, especially at 10%, showing values around 270–290 mg/100 g for K, 90–95 mg/100g for Ca, 40–50 mg/100 g for Mg, and 15–18 mg/100 g for Fe. 

The pH value and acidity are also relevant factors to determine the quality of breads and may influence the sensory properties since both could have an impact on the taste of the final product as well as on the texture, with the solubility of gluten constituent proteins being greater at acidic pH values [18]. Table 1 shows pH values between 5.20 and 5.62 for wheat breads and 5.18 and 5.54 for spelt breads, being significantly lower in breads with a higher concentration of saffron floral by-products with respect to the control samples. At the same time, acidity was higher in bread formulations with higher amount of flowers with respect to the control samples, which is related to the concentration of organic acids in the saffron floral ingredient. Acidity values ranged from 0.09 to 0.24% citric acid in all wheat and spelt bread formulations.

#### 3.1.2. Organic Acids, Soluble Sugars, and Inulin Content

The study of the composition of organic acids is an important quality parameter since they have a considerable effect on the technological characteristics, relevant sensory properties, and shelf life of breads. Data on the organic acid composition of bread formulations are shown in Table 3. Important organic acids were found in the different bread formulations, not only due to their presence in wheat and spelt flour as well as in saffron floral by-products but also as a result of the fermentation process during bread making. In the course of the fermentation period, the *Saccharomyces cerevisiae* yeast used in the formulation, together with the microbiota (bacteria and yeasts) naturally present in wheat and spelt flour, played an important role in terms of bread characteristics [19]. The biochemical changes due to the effects of fermentation on the degradation of carbohydrates and proteins mainly by the yeast and lactic acid bacteria led to the production of organic acids and other metabolites. Lactic acid was one of the main organic acids present in all bread formulations, being significantly higher in breads with 10% of saffron floral by-products (595 ± 20 and 676 ± 11 mg/100 g for WB10% and SB10%, respectively) compared to the other samples. Malic and citric acids were also found in wheat and spelt formulations in high amounts, and previous studies have reported their presence in saffron tepals [4] and in wheat flour [20,21]. Statistically significant differences were found in the content of citric acid in wheat and spelt bread formulations, being higher in WB10% (1957 ± 19 mg/100 g) and SB10% (1934 ± 22 mg/100 g) compared to the control samples, WB0% (1878 ± 7 mg/100 g) and SB0% (1600 ± 24 mg/100 g). Oxalic and fumaric acids were also found in lower amounts in the wheat and spelt bread samples, showing values around 0.24–1.98 mg/100 g for fumaric acid and 9.77–14.24 mg/100 g for oxalic acid. These results were in accordance with other studies that reported the presence of lactic, malic, and fumaric acids in wheat and spelt flours [21]. 

Furthermore, propionic acid was identified in wheat and spelt bread samples enriched with saffron flowers; this may have been generated as a result of the metabolic activity of microbiota that could metabolize molecules present in saffron floral by-products, producing different compounds. Moreover, the differences in the composition of organic acids between spelt and wheat breads could be related to the wide diversity in the presence of diverse microbial strains leading to the production of different enzymes and metabolites [22]. Therefore, the presence of organic acids in the different bread formulations could act as natural preservatives to improve their shelf life, avoiding microbiological spoilage. 

The evaluation of sugar content in bakery products such as bread is essential due to its functional role. The natural composition of sugars in the initial ingredients is a major contributor to flavor in the final product by interacting with other ingredients due to two processes which are involved: fermentation and crust browning (caramelization and the Maillard reaction) during baking. Then, the sugars remaining after fermentation also contribute to the overall color and texture of the final product, since the affinity of sugars to bind to water will delay the development of gluten, which is essential for maintaining a tender bread texture [23]. The results of the analysis of the individual sugars are shown in Table 3. 

Glucose was the major component in all bread formulations, followed by fructose and maltose. The disaccharide maltose is the first product released by the digestion of flour starch by the amylase enzyme that is further hydrolyzed to release glucose. Therefore, maltose is found in lower concentrations than glucose in the final product, ranging from 30 to 37 mg/100 g in all wheat and spelt breads. However, the glucose content is very high, showing statistically significant differences between each wheat and spelt formulation. The highest concentrations of this monosaccharide were found for WB10% (1917 ± 34 mg/100 g) and for SB10% (1829 ± 35 mg/100 g) and the lowest concentrations were found for the control samples, WB0% and SB0% (1303 ± 12 and 1210 ± 62 mg/100 g, respectively). These results were in accordance with other studies that reported the presence of maltose and glucose in wheat and spelt flours [21]. 

Fructose could be also released by hydrolysis of fructans, but this compound was only present in wheat and spelt bread formulations with 5 and 10% of saffron floral by-products. Thus, the content of this monosaccharide could be due to its natural occurrence in the saffron flowers ingredient [4]. Furthermore, the content of inulin as part of soluble dietary fiber was studied. The inulin concentration significantly increased with the content of saffron floral by-products in wheat and spelt breads. The highest amounts of inulin were present in WB10% and SB10%, with values around 6511 and 6846 mg/100 g, respectively. However, the lowest concentrations were found in samples without saffron floral by-products, being these amounts those present in wheat and spelt flours (around 5011 and 5464 mg/100 g for WB0% and SB0%, respectively). Therefore, this vegan ingredient is a natural source of dietary fiber to develop new bakery products with improved nutritional properties since inulin can be used as a prebiotic, stimulating the proliferation of the intestinal microbiota [24] At the same time, inulin contributed to an increase in TSS, since bread formulations with the highest inulin concentration (WB10% and SB10%) presented significantly higher TSS than the control samples, with values around 1.30 °Brix compared to WB0% and SB0% (0.77–0.93 °Brix) (Table 1). Moreover, the increase in TSS could be due to the presence of fructose in the composition of inulin in the WB10% and SB10% samples. 

#### 3.1.3. Texture

The textural properties are one of the quality parameters that have a crucial influence on the bread quality since they determine the shelf life and consumers’ acceptability. Table 4 shows the textural parameters (hardness, cohesiveness, springiness, gumminess, chewiness) of the different wheat and spelt bread formulations. The results show that the control wheat and spelt breads, WB0% and SB0%, were characterized by the lowest hardness, whereas the bread enrichment with saffron floral by-products resulted in a statistically significant increase in this parameter, being around 28% in WB10% and around 50% in SB10% with respect to the control samples. All wheat- and spelt-enriched bread formulations presented a statistically significant decrease in cohesiveness when compared to control breads. Thus, these results revealed that the addition of saffron floral by-products significantly affected the textural properties of breads, which could be related to the interruption of the gluten network that leads to a low cohesiveness and the crumb disintegration [7]. These findings agree with those obtained in previous studies in which bread was enriched by adding chickpea and soy flour, also observing an increase in hardness [25].

The springiness parameter is associated with freshness, and high values are desired to extend the bread shelf life. However, the enriched bread samples had a statistically significant decrease in springiness values when compared to the controls, being linked to a high crumb brittleness [26]. Chewiness and gumminess increased significantly with the saffron floral by products’ incorporation in wheat and spelt breads when compared to control samples. Similar results were found by García-Segovia, Igual and Martínez-Monzó [11] using pea protein or insect powder in the formulation of breads. These values might be related to the dietary fiber content, like inulin, which results in a strong chewiness and high water-swelling capacity [26].

#### 3.1.4. Color

Bread crumb and crust color were also evaluated since it is an important factor for the consumers’ choice of bread (Table 5). The L* values describing the brightness of breads decreased in bread crust and crumb prepared with saffron floral by-products. 

Thus, the enrichment of the bread decreased crust and crumb L* values by about 13% and 33%, respectively, in WB10% compared to the control samples, WB0%. A similar tendency was followed by spelt breads, showing a darker color of the bread crumb and crust supplemented with saffron floral by-products, decreasing the crust and crumb L* values by about 33% and 25%, respectively, in SB10% when compared to SB0%. 

**Figure 2:**
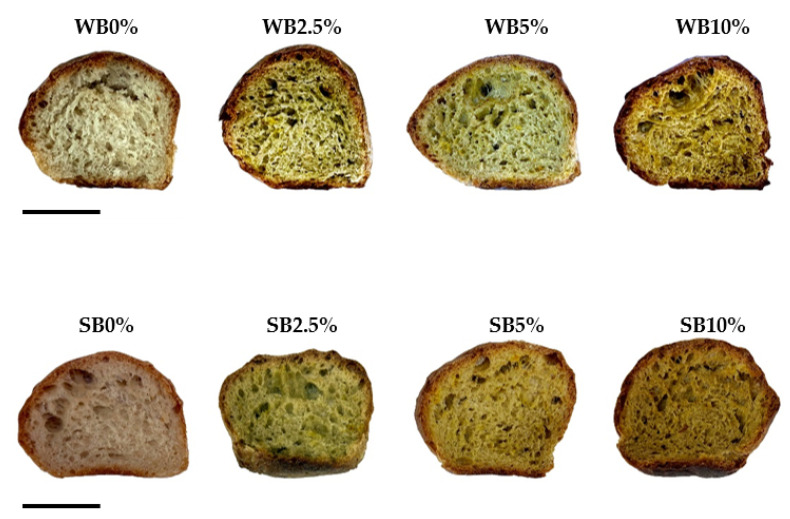
Wheat and spelt bread formulations obtained by adding different concentrations of dried saffron floral by-products. WB0%, WB2.5%, WB5%, and WB10%: wheat bread formulations with the addition of 0%, 2.5%, 5%, and 10% of dried saffron floral by-products, respectively; SB0%, SB2.5%, SB5%, and SB10%: spelt bread formulations with the addition of 0%, 2.5%, 5%, and 10% of dried saffron floral by-products, respectively. Scale bar: 2.5 cm.

These results suggested that the reduction of crust L* values could be related to an increase in Maillard browning reactions, and the decrease in L* crumb values may be explained by the intrinsic color of saffron floral by-products. Other authors reported similar observations in breads enriched with vegetal by-products [27]. Regarding the parameters, a* and b*, the crust bread showed similar values in enriched wheat and spelt bread formulations with respect to control samples. Nevertheless, the a* and b* values of the bread crumb increased in the wheat and spelt formulations with the addition of saffron flowers, resulting in redder-yellower loafs (Figure 2). Therefore, the color parameters of the crumb are related to those of the saffron flower by-products powder since the temperatures inside the loaves during baking did not exceed 100 °C [3,11].

### 3.2. Antioxidant Properties and Total Phenolic Content of Bread Formulations Enriched with Saffron Floral By-Products during Oral and Gastrointestinal In Vitro Digestion

The effect of the incorporation of saffron floral by-products into wheat and spelt bread matrices was also evaluated through the determination of the antioxidant activity and bioactive content at individual stages of simulated oral and gastrointestinal in vitro digestion. Plant bioactive compounds are very sensitive to several environmental factors, which means that their biological properties, such as the antioxidant power, could change significantly depending on the environmental conditions (pH, presence of digestive enzymes, temperature, etc.) [28]. Therefore, the changes in the antioxidant activity and in the total phenolic content at the different stages of in vitro digestion (oral, gastric, intestinal) of breads enriched with saffron floral by-products, as a rich source of phenolic compounds, were investigated.

The results of antioxidant activity (ABTS^+^ and FRAP assays) and total phenolic compounds (TPCs) in the enriched wheat and spelt bread formulations at various stages of simulated in vitro digestion are presented in Table 6. 

According to the values obtained for the ability to reduce free radicals by the ABTS^+^ assay, Fe^2+^ ions by the FRAP assay, and TPC during the in vitro digestion, the addition of saffron floral by-products into wheat and spelt breads significantly improved the phenolic content and their antioxidant ability, especially in the formulations with 10% of the saffron floral ingredient. The enriched breads, WB10% and SB10%, exhibited a significantly higher antioxidant activity (ABTS and FRAP assays) compared to the control in all digestion phases. This could be due to a significant increase of phenolic compounds from saffron floral by-products with well-known antioxidant capacities. For the ABTS assay, the antioxidant capacity increased in all bread formulations in each digestion phase, reaching the highest values under intestinal digestion conditions, being the maximum values for WB10% and SB10% after 2 h of intestinal digestion (1576 ± 34 mmol Trolox/100 g and 1583 ± 83 mmol Trolox/100 g, respectively). This increase could be due to intestinal conditions (neutral pH; presence of pancreatic enzymes and bile salts) which would facilitate an improvement in the release of bioactive compounds that may previously be bound or insolubilized due to their interaction with the bread matrix. These findings were in line with literature reports indicating that the free radical scavenging activity grew along with the increase of plant ingredients addition and with the progress of the in vitro digestion [12,28]. 

For the FRAP assay, however, the maximum values were obtained after simulated oral digestion, which remained stable after simulated gastric digestion but decreased after the intestinal phase in all bread formulations. The lowest activity after the gastric digestion was detected in control samples and the highest in the samples obtained from wheat and spelt bread with 10% of saffron floral ingredients. Therefore, the influence of the bread food matrix and its interaction with bioactive compounds from saffron flower ingredients could have played an important role in the antioxidant activity of the bread along the oral and gastrointestinal in vitro digestion.

Regarding TPC values, in the wheat and spelt bread formulations, their concentrations remained stable in the oral, gastric, and intestinal digestion steps, reaching the statistically significant highest TPC content for the bread formulations, WB10% and SB10%. In spelt breads, a slight increase in TPC after gastric conditions was observed, which could be related to an increase in the solubility of certain phenolic compounds under acidic pH conditions, which may have been previously chelated, or in reduced form [29]. It should be noted that the TPC values were high after 2 h of the intestinal stage (220 ± 17 and 251 ± 17 mg GAE/100 g for WB10% and SB10%, respectively), so that the matrix enables these bioactive compounds to reach the colon after digestion and contribute to the maintenance of intestinal health [30]. However, further research is necessary to understand their mechanism of action at the physiological level in human health, since the bioavailability of phenolic compounds through the diet depends on many factors [28].

According to the results, saffron floral by-products have a high content of phenolic compounds that remained stable in the cereal food matrix during the in vitro digestion, so they could be absorbed and exert their beneficial physiological effects. In addition, these functional breads have improved antioxidant properties, especially in the formulations with a higher content of saffron floral by-products (5 and 10%). 

**Figure 3:**
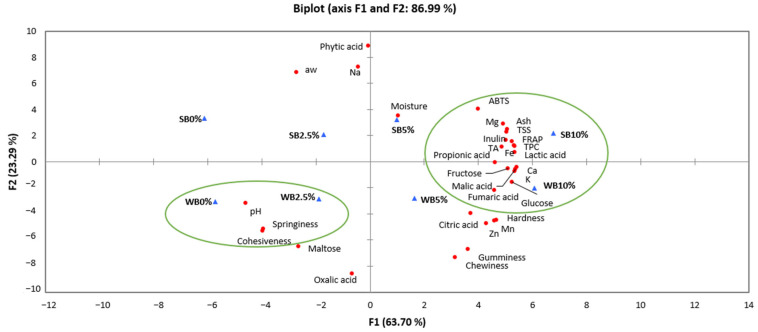
PCA biplot graph regarding physicochemical properties, minerals, organic acids, sugars content, texture, and antioxidant capacity (FRAP, ABTS) and Total Phenolic Content (TPC) after the in vitro digestion. TA: titratable acidity; TSS: total soluble sugars; WB0%, WB2.5%, WB5%, and WB10%: wheat bread formulations with the addition of 0%, 2.5%, 5%, and 10% of dried saffron floral by-products, respectively; SB0%, SB2.5%, SB5%, and SB10%: spelt bread formulations with the addition of 0%, 2.5%, 5%, and 10% of dried saffron floral by-products, respectively.

The Principal Component Analysis (PCA) biplot graph (Figure 3) showed two different groups, one related to wheat and spelt bread formulations incorporating 10% of saffron floral by-products, WB10%, and SB10%, associated with a greater antioxidant power, revealed by FRAP and ABTS assays, after the in vitro digestion process, and with a higher total phenolic content. Therefore, the cereal matrix protected the bioactive content of saffron floral by-products, avoiding their oxidation and degradation and maintaining the antioxidant activity throughout the oral and gastrointestinal in vitro digestion. It is also observed in the figure that these samples were related to a high concentration of organic acids, inulin, and minerals improving the nutritional value when compared to SB0% and SB2.5%, which were not associated with any specific characteristic. The other group, WB0% and WB2.5%, was associated with pH, cohesiveness, and springiness.

### 3.3. Sensory Analysis of Bread Formulations Enriched with Saffron Floral By-Products 

**Figure 4:**
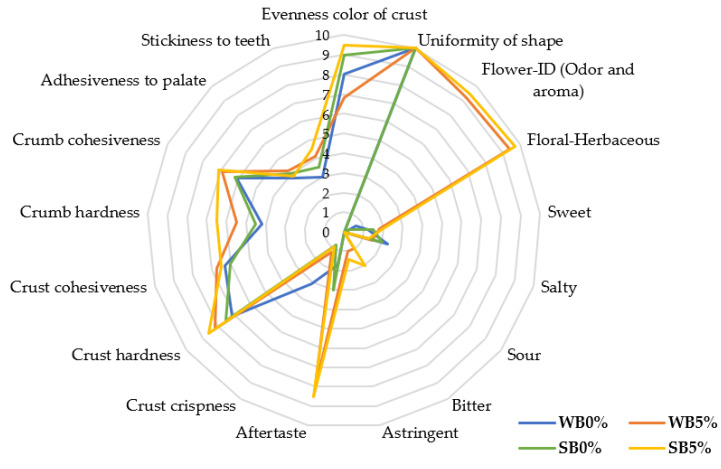
Results of the sensory analysis of selected bread formulations. Data are represented as means ± SD of three replicates. WB0% and WB5%: wheat bread formulations with the addition of 0% and 5% of dried saffron floral by-products, respectively; SB0% and SB5%: spelt bread formulations with the addition of 0% and 5% of dried saffron floral by-products, respectively.

Sensory evaluation plays an important role in the development of innovative and novel functional food products. Figure 4 summarizes the results of the sensory analysis of enriched bread formulations incorporating 5% of saffron floral by-products compared to the control samples. Appearance descriptors showed high scores regarding the uniformity of shape in all the formulations, but for the evenness of color of crust, as expected, enriched formulations such as WB5% and SB5% exhibited lower values when compared to the control samples WB0% and SB0%, respectively, due to the incorporation of saffron flowers which had an impact on the color of the crust. 

As regards flavor evaluation, WB5% and SB5% had slightly higher values in terms of sweetness, probably due to the sugar concentration of saffron flowers. In the same way, flower-ID and floral–herbaceous attributes were related to the *Crocus sativus* L. flowers in enriched bread formulations, WB5% and SB5%. The intensity of astringency and bitterness, as expected, was higher in samples formulated with saffron floral by-products because of the content of polyphenolic compounds like anthocyanins. The astringency and bitterness could be derived from the precipitation of proline-rich salivary protein in the mouth caused by phenolic compounds [31,32]. Moreover, aftertaste may be linked to the high astringency of samples.

Moreover, the results of texture attributes were in line with the TPA analysis, according to which WB5% and SB5% had higher hardness than the control samples. The use of saffron floral by-products made breads softer than the control samples, reducing their crispness and affecting their cohesiveness, probably due to the addition of this vegetal ingredient that interrupts the gluten network, making breads more fragile [33]. However, with respect to adhesiveness to palate and stickiness to teeth, no important differences were observed between the studied bread formulations. 

## 4. Conclusions

The present study aimed to evaluate the possibility of incorporating saffron floral by-products obtained from saffron spice production into wheat and spelt breads and to study their effect on physicochemical, functional, technological, and sensory properties. The enrichment of traditional breads with saffron flowers was an effective tool that allowed for obtaining functional food with significantly enhanced nutritional and functional potential, increasing the content of dietary fiber such as inulin, minerals, and organic acids, and showing a high antioxidant capacity and total phenolic content after the in vitro digestion process. Therefore, this study confirms that saffron floral by-products are a sustainable alternative for the development of novel bakery products suitable for vegans and vegetarians which could exert beneficial effects on human health after their intake. It also encompasses the valorization of a biomass that is currently unexploited, minimizing, at the same time, its environmental impact. Furthermore, this research provided new information for further studies that focus on the effects of the saffron floral ingredient at a physiological level after the intake of these functional breads.

## Figures and Tables

**Table 1:** Physicochemical parameters of the different wheat and spelt bread formulations enriched with saffron floral by-products (moisture, ash, pH, acidity, TSS, aw) ^1^.

	Moisture (%)	Ash (%)	pH	TA (% Citric Acid)	Aw	TSS (°Brix)
WB0%	31.12 ± 2.43 ^ab^	2.75 ± 0.07 ^c^	5.62 ± 0.01 ^a^	0.09 ± 0.01 ^d^	0.85 ± 0.03	0.77 ± 0.01 ^d^
WB2.5%	25.47 ± 2.49 ^b^	2.95 ± 0.17 ^c^	5.50 ± 0.01 ^b^	0.12 ± 0.01 ^c^	0.84 ± 0.02	0.93 ± 0.02 ^c^
WB5%	31.12 ± 2.40 ^ab^	3.44 ± 0.10 ^b^	5.31 ± 0.02 ^c^	0.23 ± 0.02 ^b^	0.84 ± 0.04	1.07 ± 0.02 ^b^
WB10%	32.44 ± 2.44 ^a^	3.79 ± 0.12 ^a^	5.20 ± 0.01 ^d^	0.28 ± 0.02 ^a^	0.84 ± 0.02	1.30 ± 0.01 ^a^
SB0%	32.71 ± 2.61	3.05 ± 0.15 ^c^	5.54 ± 0.01 ^a^	0.10 ± 0.02 ^b^	0.86 ± 0.01	0.97 ± 0.01 ^d^
SB2.5%	29.51 ± 2.46	3.37 ± 0.11 ^b^	5.23 ± 0.00 ^b^	0.21 ± 0.01 ^a^	0.85 ± 0.00	1.03 ± 0.03 ^c^
SB5%	31.89 ± 2.36	3.39 ± 0.08 ^b^	5.21 ± 0.00 ^c^	0.23 ± 0.02 ^a^	0.85 ± 0.01	1.07 ± 0.02 ^b^
SB10%	31.50 ± 2.33	3.95 ± 0.16 ^a^	5.18 ± 0.01 ^d^	0.24 ± 0.01 ^a^	0.85 ± 0.03	1.30 ± 0.01 ^a^

^1^ Means ± standard deviation in the same column followed by different lowercase letters indicate statistically significant differences at (*p* ≤ 0.05) for each wheat or spelt sample (*n* = 3); TA: titratable acidity; TSS: total soluble sugars; WB0%, WB2.5%, WB5%, and WB10%: wheat bread formulations with the addition of 0%, 2.5%, 5%, and 10% of dried saffron floral by-products, respectively; SB0%, SB2.5%, SB5%, and SB10%: spelt bread formulations with the addition of 0%, 2.5%, 5%, and 10% of dried saffron floral by-products, respectively.

**Table 2:** Composition of minerals (mg/100 g dw) of the different wheat and spelt bread formulations enriched with saffron floral by-products ^1^.

		WB0%	WB2.5%	WB5%	WB10%	SB0%	SB2.5%	SB5%	SB10%
Macrominerals	Ca	39.22 ± 1.24 ^b^	56.18 ± 5.15 ^b^	67.92 ± 8.02 ^a^	91.63 ± 10.02 ^a^	33.47 ± 6.00 ^c^	52.74 ± 8.10 ^bc^	65.98 ± 1.33 ^b^	94.56 ± 3.14 ^a^
	K	162 ± 3 ^d^	196 ± 9 ^c^	226 ± 8 ^b^	277 ± 14 ^a^	153 ± 10 ^c^	178 ± 11 ^b^	209 ± 16 ^b^	289 ± 18 ^a^
	Mg	31.21 ± 1.54^c^	34.72 ± 1.82 ^bc^	36.97 ± 1.43 ^b^	41.61 ± 1.93 ^a^	33.95 ± 3.01 ^b^	34.46 ± 3.11 ^b^	40.81 ± 1.04 ^a^	48.46 ± 8.03 ^a^
	Na	641 ± 18 ^ab^	713 ± 32 ^a^	682 ± 23 ^ab^	605 ± 85 ^b^	810 ± 55	713 ± 55	743 ± 13	739 ± 12
Microminerals	Fe	2.01 ± 0.10 ^c^	6.01 ± 1.15 ^b^	9.15 ± 1.96 ^b^	15.87 ± 1.25 ^a^	3.27 ± 1.83 ^c^	6.27 ± 0.35 ^c^	10.63 ± 0.39 ^b^	17.85 ± 0.40 ^a^
	Mn	1.03 ± 0.19 ^b^	1.22 ± 0.03 ^ab^	1.25 ± 0.05 ^ab^	1.42 ± 0.21 ^a^	0.74 ± 0.09 ^b^	0.91 ± 0.10 ^ab^	1.04 ± 0.01 ^ab^	1.37 ± 0.20 ^a^
	Zn	0.77 ± 0.11	0.97 ± 0.00	0.97 ± 0.01	1.03 ± 0.06	0.71 ± 0.02	0.78 ± 0.06	0.81 ± 0.10	0.96 ± 0.20

^1^ Means ± standard deviation in the same row followed by different lowercase letters indicate statistically significant differences at (*p* ≤ 0.05) for each wheat or spelt sample (*n* = 3); WB0%, WB2.5%, WB5%, and WB10%: wheat bread formulations with the addition of 0%, 2.5%, 5%, and 10% of dried saffron floral by-products, respectively; SB0%, SB2.5%, SB5%, and SB10%: spelt bread formulations with the addition of 0%, 2.5%, 5%, and 10% of dried saffron floral by-products, respectively.

**Table 3:** Content of organic acids, soluble sugars, and inulin (mg/100 g dw) of the different wheat and spelt bread formulations enriched with saffron floral by-products ^1^.

		WB0%	WB2.5%	WB5%	WB10%	SB0%	SB2.5%	SB5%	SB10%
Organic acids	Phytic acid	n.d.	n.d.	n.d.	n.d.	178 ± 27	165 ± 5	168 ± 11	172 ± 3
	Lactic acid	241 ± 60 ^d^	384 ± 22 ^c^	503 ± 19 ^b^	595 ± 20 ^a^	301 ± 33 ^d^	385 ± 6 ^c^	448 ± 5 ^b^	676 ± 11 ^a^
	Citric acid	1878 ± 7 ^c^	1895 ± 6 ^bc^	1922 ± 14 ^b^	1957 ± 19 ^a^	1600 ± 24 ^c^	1885 ± 5 ^b^	1910 ± 9 ^b^	1934 ± 22 ^a^
	Malic acid	132 ± 5 ^d^	214 ± 11 ^c^	335 ± 30 ^b^	516 ± 30 ^a^	102 ± 10 ^d^	189 ± 16 ^c^	292 ± 26 ^b^	521 ± 32 ^a^
	Oxalic acid	14.24 ± 1.90	13.91 ± 0.62	13.84 ± 0.93	13.79 ± 0.93	11.33 ± 0.76	11.75 ± 0.22	9.77 ± 3.73	10.98 ± 0.26
	Fumaric acid	0.24 ± 0.07 ^c^	0.04 ± 0.00 ^c^	1.05 ± 0.08 ^b^	1.98 ± 0.13 ^a^	n.d.	n.d.	n.d.	1.71 ± 0.20
	Propionic acid	n.d.	835 ± 71 ^b^	933 ± 33 ^a^	982 ± 48 ^a^	n.d.	845 ± 9 ^c^	910 ± 16 ^b^	1051 ± 48 ^a^
Soluble sugars	Glucose	1303 ± 12 ^d^	1411 ± 16 ^c^	1617 ± 13 ^b^	1917 ± 34 ^a^	1210 ± 62 ^d^	1317 ± 25 ^c^	1512 ± 82 ^b^	1829 ± 35 ^a^
	Maltose	37.70 ± 0.14 ^a^	33.32 ± 0.10 ^c^	36.75 ± 0.16 ^b^	33.11 ± 0.33 ^c^	33.19 ± 0.24 ^a^	33.03 ± 0.41 ^a^	31.19 ± 0.32 ^b^	30.49 ± 0.13 ^b^
	Fructose	n.d.	n.d.	480 ± 40 ^b^	913 ± 82 ^a^	n.d.	n.d.	307 ± 48 ^b^	813 ± 12 ^a^
	Inulin	5011 ± 70 ^c^	5377 ± 95 ^b^	6504 ± 58 ^a^	6511 ± 223 ^a^	5464 ± 80 ^c^	5682 ± 112 ^c^	6231 ± 211 ^b^	6846 ± 48 ^a^

^1^ Means ± standard deviation in the same row followed by different lowercase letters indicate statistically significant differences at (*p* ≤ 0.05) for each wheat or spelt sample (*n* = 3); WB0%, WB2.5%, WB5%, and WB10%: wheat bread formulations with the addition of 0%, 2.5%, 5%, and 10% of dried saffron floral by-products, respectively; SB0%, SB2.5%, SB5%, and SB10%: spelt bread formulations with the addition of 0%, 2.5%, 5%, and 10% of dried saffron floral by-products, respectively; n.d.: not detected.

**Table 4:** Parameters of the texture of the different wheat and spelt bread formulations enriched with saffron floral by-products ^1^.

	Hardness (N)	Cohesiveness	Springiness (mm)	Gumminess (N)	Chewiness (N)
WB0%	42.24 ± 2.45 ^d^	0.93 ± 0.02 ^a^	1.59 ± 0.01 ^a^	39.47 ± 0.41 ^d^	37.73 ± 0.74 ^c^
WB2.5%	48.36 ± 3.32 ^c^	0.88 ± 0.00 ^b^	1.50 ± 0.08 ^b^	42.58 ± 0.67 ^c^	40.00 ± 0.49 ^b^
WB5%	51.12 ± 1.12 ^b^	0.87 ± 0.01 ^b^	1.49 ± 0.03 ^b^	44.68 ± 0.48 ^b^	41.51 ± 0.23 ^b^
WB10%	54.17 ± 0.53 ^a^	0.86 ± 0.00 ^b^	1.47 ± 0.02 ^c^	46.91± 0.82 ^a^	43.21 ± 0.15 ^a^
SB0%	34.65 ± 1.66 ^c^	0.89 ± 0.01 ^a^	1.53 ± 0.04 ^a^	31.14 ± 1.11 ^d^	28.64 ± 0.16 ^c^
SB2.5%	41.31 ± 2.24 ^b^	0.84 ± 0.02 ^b^	1.43 ± 0.00 ^b^	34.76 ± 0.53 ^c^	31.93 ± 0.27 ^b^
SB5%	43.75 ± 2.35 ^b^	0.82 ± 0.02 ^bc^	1.40 ± 0.00 ^c^	36.06 ± 0.14 ^b^	32.94 ± 0.98 ^b^
SB10%	52.12 ± 3.87 ^a^	0.80 ± 0.00 ^c^	1.37 ± 0.01 ^d^	41.91 ± 1.14 ^a^	38.22 ± 0.54 ^a^

^1^ Means ± standard deviation in the same column followed by different lowercase letters indicate statistically significant differences at (*p* ≤ 0.05) for each wheat or spelt sample (*n* = 3); WB0%, WB2.5%, WB5%, and WB10%: wheat bread formulations with the addition of 0%, 2.5%, 5%, and 10% of dried saffron floral by-products, respectively; SB0%, SB2.5%, SB5%, and SB10%: spelt bread formulations with the addition of 0%, 2.5%, 5%, and 10% of dried saffron floral by-products, respectively.

**Table 5:** Color of the crust and crumb of the different wheat and spelt bread formulations enriched with saffron floral by-products ^1^.

	Crust			Crumb		
	L*	a*	b*	L*	a*	b*
WB0%	40.25 ± 3.15	12.49 ± 0.19 ^a^	22.22 ± 2.19 ^b^	61.86 ± 1.69 ^a^	0.28 ± 0.10 ^b^	18.45 ± 0.35 ^b^
WB2.5%	37.21 ± 2.36	11.35 ± 0.63 ^ab^	20.18 ± 2.51 ^b^	51.72 ± 3.73 ^b^	−0.38 ± 0.02 ^c^	27.83 ± 0.60 ^a^
WB5%	41.95 ± 2.37	10.72 ± 0.47 ^b^	22.99 ± 1.56 ^a^	47.72 ± 0.63 ^b^	0.08 ± 0.04 ^b^	27.44 ± 3.54 ^a^
WB10%	34.78 ± 3.56	9.21 ± 0.53 ^c^	17.05 ± 2.32 ^b^	41.29 ± 1.47 ^c^	1.52 ± 0.32 ^a^	28.35 ± 1.89 ^a^
SB0%	50.58 ± 1.71 ^a^	11.05 ± 0.34 ^a^	25.35 ± 1.16 ^a^	63.15 ± 5.68 ^a^	0.02 ± 0.00 ^d^	19.08 ± 1.07 ^b^
SB2.5%	43.54 ± 5.48 ^ab^	10.60 ± 1.15 ^a^	23.30 ± 2.87 ^a^	58.45 ± 1.81 ^ab^	0.30 ± 0.01 ^c^	28.21 ± 1.40 ^a^
SB5%	40.15 ± 0.42 ^bc^	11.06 ± 0.56 ^a^	21.42 ± 0.57 ^a^	52.50 ± 2.54 ^c^	0.59 ± 0.03 ^b^	30.70 ± 0.81 ^a^
SB10%	33.75 ± 1.42 ^c^	8.73 ± 0.53 ^b^	15.22 ± 1.14 ^b^	47.01 ± 3.63 ^c^	1.75 ± 0.09 ^a^	30.57 ± 1.70 ^a^

^1^ Means ± standard deviation in the same column followed by different lowercase letters indicate statistically significant differences at (*p* ≤ 0.05) for each wheat or spelt sample (*n* = 3); WB0%, WB2.5%, WB5%, and WB10%: wheat bread formulations with the addition of 0%, 2.5%, 5%, and 10% of dried saffron floral by-products, respectively; SB0%, SB2.5%, SB5%, and SB10%: spelt bread formulations with the addition of 0%, 2.5%, 5%, and 10% of dried saffron floral by-products, respectively.

**Table 6:** Antioxidant properties (FRAP and ABTS assays) and Total Phenolic Content (TPC) of the different wheat and spelt bread formulations enriched with saffron floral by-products during the oral and gastrointestinal in vitro digestion process ^1^.

		In Vitro Digestion			
		Oral	Gastric	Intestinal (1 h)	Intestinal (2 h)
ABTS (mmol Trolox/100 g)	WB0%	3.47 ± 1.75	22.37 ± 1.99 ^c^	421 ± 12 ^a^	458 ± 6 ^b^
	WB2.5%	3.94 ± 1.28	34.32 ± 8.47 ^b^	437 ± 4 ^a^	452 ± 10 ^b^
	WB5%	3.61 ± 2.61	48.74 ± 2.14 ^a^	435 ± 8 ^a^	461 ± 5 ^b^
	WB10%	4.94 ± 1.50	49.99 ± 0.98 ^a^	399 ± 7 ^b^	1576 ± 34 ^a^
	SB0%	2.20 ± 0.16 ^b^	30.74 ± 3.31 ^b^	429 ± 11	437± 27 ^b^
	SB2.5%	5.49 ± 0.67 ^a^	43.34 ± 1.99 ^a^	430 ± 12	1373 ± 59 ^a^
	SB5%	4.91 ± 0.81 ^a^	45.44 ± 6.40 ^a^	426 ± 19	1458± 177 ^a^
	SB10%	5.69 ± 0.20 ^a^	50.54 ± 0.39 ^a^	437 ± 27	1583 ± 83 ^a^
FRAP (mmol Trolox/100 g)	WB0%	0.89 ± 0.50 ^c^	8.07 ± 2.03 ^c^	n.d.	n.d.
	WB2.5%	14.09 ± 4.08 ^b^	17.12 ± 0.56 ^bc^	1.40 ± 0.83 ^b^	2.43 ± 0.73 ^b^
	WB5%	28.05 ± 4.85 ^a^	25.64 ± 6.04 ^b^	9.83 ± 5.35 ^ab^	15.00 ± 6.58 ^ab^
	WB10%	38.77 ± 5.90 ^a^	37.67 ± 4.67 ^a^	20.71 ± 7.96 ^a^	27.89 ± 6.99 ^a^
	SB0%	6.99 ± 0.73 ^c^	11.21 ± 1.01 ^b^	n.d.	n.d.
	SB2.5%	18.08 ± 4.03 ^bc^	21.56 ± 1.19 ^b^	3.97 ± 2.81 ^b^	7.60 ± 0.34 ^c^
	SB5%	32.01 ± 9.90 ^b^	26.69 ± 12.58 ^ab^	14.26 ± 8.84 ^b^	18.16 ± 4.89 ^b^
	SB10%	61.86 ± 10.75 ^a^	43.97 ± 6.96 ^a^	31.79 ± 1.22 ^a^	36.62 ± 3.130 ^a^
TPC (mg GAE/100 g)	WB0%	76.99 ± 6.60 ^d^	118 ± 8 ^d^	109 ± 7 ^d^	91.31 ± 5.25 ^c^
	WB2.5%	111 ± 6 ^c^	149 ± 16 ^c^	140 ± 16 ^c^	146 ± 13 ^b^
	WB5%	198 ± 8 ^b^	207 ± 1 ^b^	222 ± 8 ^b^	166 ± 14 ^b^
	WB10%	221 ± 18 ^a^	231 ± 11 ^a^	272 ± 17 ^a^	220 ± 17 ^a^
	SB0%	102 ± 16 ^c^	181 ± 92	137 ± 18 ^c^	105 ± 15 ^c^
	SB2.5%	124 ± 32 ^bc^	236 ± 129	135 ± 10 ^c^	135 ± 45 ^bc^
	SB5%	175 ± 1 ^b^	251 ± 71	187 ± 6 ^b^	186 ± 5 ^b^
	SB10%	261 ± 13 ^a^	313 ± 63	258 ± 25 ^a^	251 ± 17 ^a^

^1^ Means ± standard deviation in the same column followed by different lowercase letters indicate statistically significant differences at (*p* ≤ 0.05) for each wheat or spelt sample (*n* = 3); WB0%, WB2.5%, WB5%, and WB10%: wheat bread formulations with the addition of 0%, 2.5%, 5%, and 10% of dried saffron floral by-products, respectively; SB0%, SB2.5%, SB5%, and SB10%: spelt bread formulations with the addition of 0%, 2.5%, 5%, and 10% of dried saffron floral by-products, respectively; n.d.: not detected.

## Data Availability

Data is contained within the article or supplementary material.

## Supplementary Materials

The following supporting information can be downloaded at: https://www.mdpi.com/article/10.3390/foods12122380/s1; Table S1: Lexicon for the sensory description of bread formulations.

## References

[1] Tabanelli G., Pasini F., Riciputi Y., Vannini L., Gozzi G., Balestra F., Caboni M.F., Gardini F., Montanari C. (2018). Fermented nut-based vegan food: Characterization of a home made product and scale-up to an industrial pilot-scale production. J. Food Sci..

[2] Sun C., Ge J., He J., Gan R., Fang Y. (2021). Processing, quality, safety, and acceptance of meat analogue products. Engineering.

[3] Cerdá-Bernad D., Clemente-Villalba J., Valero-Cases E., Pastor J.-J., Frutos M.-J. (2022). Novel insight into the volatile profile and antioxidant properties of *Crocus sativus* L. flowers. Antioxidants.

[4] Serrano-Díaz J., Sánchez A.M., Martínez-Tomé M., Winterhalter P., Alonso G.L. (2013). A contribution to nutritional studies on *Crocus sativus* flowers and their value as food. J. Food Compos. Anal..

[5] Cerdá-Bernad D., Costa L., Serra A.T., Bronze M.R., Valero-Cases E., Pérez-Llamas F., Candela M.E., Arnao M.B., Barberán F.T., Villalba R.G. (2022). Saffron against neuro-cognitive disorders: An overview of its main bioactive compounds, their metabolic fate and potential mechanisms of neurological protection. Nutrients.

[6] Dewettinck K., Van Bockstaele F., Kühne B., Van de Walle D., Courtens T.M., Gellynck X. (2008). Nutritional value of bread: Influence of processing, food interaction and consumer perception. J. Cereal Sci..

[7] Pycia K., Ivanišová E. (2020). Physicochemical and antioxidant properties of wheat bread enriched with hazelnuts and walnuts. Foods.

[8] Świeca M., Gawlik-Dziki U., Dziki D., Baraniak B. (2017). Wheat bread enriched with green coffee—In vitro bioaccessibility and bioavailability of phenolics and antioxidant activity. Food Chem..

[9] AOAC (1995). Official Methods of Analysis.

[10] Cerdá-Bernad D., Valero-Cases E., Pastor J.J., Frutos M.J., Pérez-Llamas F. (2020). Probiotic red quinoa drinks for celiacs and lactose intolerant people: Study of functional, physicochemical and probiotic properties during fermentation and gastrointestinal digestion. Int. J. Food Sci. Nutr..

[11] García-Segovia P., Igual M., Martínez-Monzó J. (2020). Physicochemical properties and consumer acceptance of bread enriched with alternative proteins. Foods.

[12] Gawlik-Dziki U., Dziki D., Baraniak B., Lin R. (2009). The effect of simulated digestion in vitro on bioactivity of wheat bread with Tartary buckwheat flavones addition. LWT—Food Sci. Technol..

[13] Sicari V., Romeo R., Mincione A., Santacaterina S., Tundis R., Loizzo M.R. (2023). Ciabatta bread incorporating goji (*Lycium barbarum* L.): A new potential functional product with impact on human health. Foods.

[14] He H., Hoseney R.C. (1990). Changes in bread firmness and moisture during long-term storage. Cereal Chem..

[15] European Food Safety Authority (2017). Dietary reference values for nutrients. EFSA J..

[16] Fahim N., Sadat S., Janati F., Feizy J. (2012). Chemical composition of agriproduct saffron (*Crocus sativus* L.) petals and its considerations as animal feed. GIDA.

[17] Fernández-Canto M.N., García-Gómez M.B., Boado-Crego S., Vázquez-Odériz M.L., Muñoz-Ferreiro M.N., Lombardero-Fernández M., Pereira-Lorenzo S., Romero-Rodríguez M.Á. (2022). Element content in different wheat flours and bread varieties. Foods.

[18] Takeda K., Matsumura Y., Shimizu M. (2001). Emulsifying and surface properties of wheat gluten under acidic conditions. J. Food Sci..

[19] Arendt E.K., Ryan L.A.M., Dal Bello F. (2007). Impact of sourdough on the texture of bread. Food Microbiol..

[20] Munch-Petersen A. (2008). Citric acid content in meal of certain cereals arid leguminous plants: A contribution to the question of exogenous citric acid. Acta Physiol. Scand..

[21] Shewry P.R., America AH P., Lovegrove A., Wood A.J., Plummer A., Evans J., van den Broeck H.C., Gilissen L., Mumm R., Ward J.L. (2022). Comparative compositions of metabolites and dietary fibre components in doughs and breads produced from bread wheat, emmer and spelt and using yeast and sourdough processes. Food Chem..

[22] De Luca L., Aiello A., Pizzolongo F., Blaiotta G., Aponte M., Romano R. (2021). Volatile organic compounds in breads prepared with different sourdoughs. Appl. Sci..

[23] Purlis E. (2010). Browning development in bakery products—A review. J. Food Eng..

[24] Kolida S., Tuohy K., Gibson G.R. (2002). Prebiotic effects of inulin and oligofructose. Br. J. Nutr..

[25] Lian H., Luo K., Gong Y., Zhang S., Serventi L. (2020). Okara flours from chickpea and soy are thickeners: Increased dough viscosity and moisture content in gluten-free bread. Int. J. Food Sci. Technol..

[26] Tóth M., Kaszab T., Meretei A. (2022). Texture profile analysis and sensory evaluation of commercially available gluten-free bread samples. Eur. Food Res. Technol..

[27] Frutos M.J., Guilabert-Antón L., Tomás-Bellido A., Hernández-Herrero J.A. (2008). Effect of artichoke (*Cynara scolymus* L.) fiber on textural and sensory qualities of wheat bread. Food Sci. Technol. Int..

[28] Czubaszek A., Czaja A., Sokół-Łętowska A., Kolniak-Ostek J., Kucharska A.Z. (2021). Changes in antioxidant properties and amounts of bioactive compounds during simulated in vitro digestion of wheat bread enriched with plant extracts. Molecules.

[29] Gião M.S., Gomes S., Madureira A.R., Faria A., Pestana D., Calhau C., Pintado M.E., Azevedo I., Malcata F.X. (2012). Effect of *in vitro* digestion upon the antioxidant capacity of aqueous extracts of *Agrimonia eupatoria*, *Rubus idaeus*, *Salvia* sp. and *Satureja montana*. Food Chem..

[30] Laparra J.M., Sanz Y. (2010). Interactions of gut microbiota with functional food components and nutraceuticals. Pharmacol. Res..

[31] Hoye J., Clifford Ross C.F. (2011). Total phenolic content, consumer acceptance, and instrumental analysis of bread made with grape seed flour. J. Food Sci..

[32] Soares S., Kohl S., Thalmann S., Mateus N., Meyerhof W., De Freitas V. (2013). Different phenolic compounds activate distinct human bitter taste receptors. J. Agric. Food Chem..

[33] Altinok E., Kurultay S., Boluk E., Atik D.S., Kopuk B., Gunes R., Palabiyik I., Konar N., Toker O.S. (2022). Investigation of using possibility of grape pomace in wafer sheet for wheat flour substitution. Int. J. Food Sci. Technol..

