# Supplementary material for: Saffron Floral By-Products as Novel Sustainable Vegan Ingredients for the Functional and Nutritional Improvement of Traditional Wheat and Spelt Breads
Source: Foods. 2023 Jun 15;12(12):2380. doi: 10.3390/foods12122380 (PMC10297254; doi:10.3390/foods12122380)
Supplement: Supplementary file 1 [file foods-12-02380-s001.zip › foods-2455456-supplementary.pdf]

Table S1. Lexicon for the sensory description of bread formulations.

	Attributes	Definition	References and intensities
Appearance	Evenness colour of crust	Distribution of colour	No uniform = 0.0; Uniform = 10.0
	Uniformity of shape	Homogeneity of the bread	No uniform = 0.0; Uniform = 10.0
Flavour	Flower *Crocus sativus* L.-ID (Odor and aroma)	Floral and herbaceous odor associated with *Crocus sativus* L. flowers	5 g of grinded dried flowers of *Crocus sativus* L. + 100 mL H_2O = 4; 20 g of grinded dried flowers of *Crocus sativus* L. + 25 mL H_2O = 10
	Floral-Herbaceous	Fresh, green, slightly sour aromatics associated with green vegetables	Kroger lima beans (canned) = 3.0; Small sprig fresh parsley = 7.0 (aroma); Fresh parsley = 10.0
	Sweet	The fundamental taste factor associated with a sucrose solution	Sucrose solution 4% = 2.5; Sucrose solution 8% = 5.0; Sucrose solution 16% = 9.5
	Salty	Fundamental taste sensation of which sodium chloride is typical	0.2% NaCl solution = 2.5; 0.35% NaCl solution = 5.0; 0.8% NaCl solution = 9.0
	Sour	The taste stimulated by acids, such as citric and malic acids	Tartaric acid solution 0.05% = 2.5; Tartaric acid solution 0.08% = 4.0; Tartaric acid solution 0.20% = 9.5
	Bitter	The taste stimulated by substances such as quinine or caffeine	Caffeine solution 0.05% = 2.5; Caffeine solution 0.08% = 4.0
	Astringent	Dry sensation on the surface of the tongue or mouth associated with alum solution	Alum solution 0.03% = 1.5; Alum solution 0.05% = 2.5; Alum solution 0.1% = 5.0
	Aftertaste	Time in which the specific flavour of bread remains in the mouth after swallowing the sample	10 s = 2.0; 30 s = 8.0
Texture	Crust crispness	The noise and force with which the sample breaks or fractured	Chewing gum (Wrigley) = 0.0; Graham cracker (Honey maid) = 5.0; Corn flakes (Kellogg's) = 10.0
	Crust hardness	Force required to bite through	Cream cheese (Philadelphia) = 1.0; Olives (Goya Foods) = 6.0; Fresh carrot = 10.0
	Crust cohesiveness	Amount of sample that deforms rather than ruptures	Corn muffin (Jiffy) = 1.0; Pretzel = 6.0; Chewing gum (Wrigley) = 10.0
	Crumb hardness	Force required to bite through	Cream cheese (Philadelphia) = 1.0; Olives (Goya Foods) = 6.0; Fresh carrot = 10.0
	Crumb cohesiveness	Amount of sample that deforms rather than ruptures	Corn muffin (Jiffy) = 1.0; Pretzel = 6.0; Chewing gum (Wrigley) = 10.0
	Adhesiveness to palate	Force required to remove sample from palate	Fresh cherry tomato = 0.0; Pretzel rod (Bachman) = 7.0; Rice Krispies (Kellogg's) = 10.0
	Stickiness to teeth	Amount of product that adheres to oral surface	Fresh cherry tomato = 0.0; Pretzel rod (Bachman) = 7.0; Rice Krispies (Kellogg's) = 10.0
